# The labor force participation of Indian women before and after widowhood

**DOI:** 10.4054/demres.2020.43.24

**Published:** 2020-09-02

**Authors:** Megan N. Reed

**Affiliations:** 1 University of Pennsylvania, Philadelphia, PA, USA.

## Abstract

**BACKGROUND:**

Due to its young age structure and taboos on widow remarriage, India has a large and relatively young female widow population. Many of India’s widows are in prime working ages. India has one of the lowest female labor force participation rates in the world.

**OBJECTIVE:**

This paper calculates the effect of widowhood on the labor force participation of Indian widows. The analysis documents how labor force participation changes associated with widowhood vary by age, caste/religion, relation to head of household, rural/urban status, and region.

**METHODS:**

Using the India Human Development Survey (IHDS), the analysis tracks 3,217 women who experience the loss of their spouse between the two survey waves. Individual fixed effects regressions are used to measure the association between the transition to widowhood and changes in the number of days worked in the past year.

**RESULTS:**

Widowhood was associated with a decrease in days worked for older women; but for women widowed before age 52, widowhood was associated with a large increase in the number of days they worked. Widows who joined the labor force were more likely to gain employment in permanent and salaried work than married women. Widows who resided with their in-laws or who became the household head after their husband’s death saw increases in their work participation whereas those who lived in households headed by their adult children experienced negative widowhood effects on their work participation.

**CONTRIBUTION:**

These findings highlight the important link between marital status and female employment in India.

## 1. Introduction

With just under a quarter of women in the labor force, India has one of the lowest rates of female labor force participation (LFP) in the world (Fletcher, Pande, and Moore 2017; Lahoti and Swaminathan 2016). Research suggests that marriage plays a role in keeping Indian women out of the labor force. Fletcher, Pande, and Moore (2017) document that the largest gaps in work participation are by marital status rather than the presence of children in the home, which is a more important factor in other social contexts. Studies on attitudes document high rates of social disapproval of women working after marriage in India and social costs on the husbands of working women (Bernhardt et al. 2018).

Though they have received little attention in the scholarship on India’s female LFP, Indian widows are an interesting exception to national patterns of low female employment. Labor force participation rates for widows in working ages are generally higher than for women of other marital statuses (Jensen 2005; Chen and Dreze 1992; Das 2015). This could be due to poverty shocks that may follow the loss of an income earner in the household. For example, a widow who did not work before her husband’s death may be expected to ‘earn her keep’ in the home of her kin or her husband’s family after her husband’s death (Chen 2000). Entering the labor force may come with social stigma for some widows, who may be expected to adhere to strict norms of social seclusion and ritual mourning after the death of their husband. The qualitative literature has documented Indian women’s experience with the both frightening and liberating process of becoming financially independent after widowhood (Chen 2000; Lamb 2000). Ambiguity remains as to whether widow employment should be celebrated or prevented given the vulnerable position of widows. Regardless, documenting the impact of widowhood on women’s work participation can expand our understanding of the linkages between marital status and women’s employment in Indian society.

It is important to recognize two important demographic facts about widows in India; first, that they are substantial in number; and second, that they are generally younger than widow populations in other countries (Lloyd-Sherlock, Corso, and Minicuci 2015). At the time of the 2011 Indian Census, there were 43 million widowed women in India, a population comparable to a mid-sized country (Government of India 2011). In fact, 7.4% of all Indian women are widows compared to the mere 1.8% of Indian men who are widowers (Government of India 2011). This disparity can, at least partially, be explained by taboos on widow remarriage, which leave most young widows in the widowed marital category for the remainder of their lives (Chen 2000; Jensen 2005). This taboo does not hold for widowers. As a result of the remarriage taboo and India’s young age pyramid, India has a relatively young widowed population. This means that, unlike in other countries, the issues associated with widowhood in India are not limited to the social protection of elderly widowed women. In fact, many widows are in prime working ages. According to the 2011 Indian Census numbers, 4.6% of women in the 35–39 age group were widowed, and by the 45–49 age group 10.8% were widows (Government of India 2011).

This paper will use panel data to describe patterns of labor force participation among Indian women who experience widowhood. These findings fill important gaps in the literature. Work participation has received little attention in the widowhood effects literature, which has primarily focused on widowhood’s impact on mortality, morbidity, and mental health (Shor et al. 2012; Elwert and Christakis 2008; Agrawal and Keshri 2014; Siflinger 2017). In addition, research on widowhood in the Indian context has come mostly from either cross-sectional national surveys or one-time qualitative or survey data collection exercises, which are not nationally representative, limiting the generalizability of findings.

This paper is the first, to the author’s knowledge, to document the effect of the shock of widowhood on women’s employment behavior in India. This is accomplished by harnessing one of the first large-scale nationally representative panel studies in India, the India Human Development Survey (IHDS), which has completed two waves so far. This large dataset includes 3,217 women who experienced the loss of their husband between the first wave of the survey in 2004–2005 and the second wave in 2011–2012, a seven-year period. The analysis will document how LFP changes associated with the widowhood shock vary by age, caste/religion, living arrangements, residence type, and region of India using individual fixed effects regressions. These findings will help illuminate why widows in India tend to have higher work participation than non-widows, what type of work they do after widowhood, and what types of women are most likely to be affected. The conclusion outlines how these results suggest larger insights into women’s relationship to the labor market in India.

## 2. Review of the literature

### 2.1 Female labor force participation and marital status in India

India’s female LFP has begun to attract the attention of academics as it appears to deviate from Goldin’s (1994) famous U-shaped pattern. Goldin (1994) documented that economic development has a U-shaped relationship to married women’s LFP across various social contexts. Initially female employment is usually high in agriculture-based economies. As the economy grows, female LFP declines because women have less access to building the human capital necessary for the new labor market. Their employment rises again as women’s education levels begin to match those of men and it becomes costly to keep them out of the labor force. Economists have noted that despite large growth in female education in India, their employment has stagnated for decades and recently started to decline, deviating from this U-shaped pattern (Lahoti and Swaminathan 2016). There are a number of factors that could be keeping Indian women out of the labor force. Family income effects are found to be an important component of why women are kept out of the labor force despite their increasing education (Chatterjee, Desai, and Vanneman 2018). Some argue that declining female employment could be due to a strong crowding-out effect for high-skilled workers, stagnating growth in the size of the white-collar sector, and a weakening of the positive effect of education on female labor force participation (Klasen and Pieters 2015). The decline in employment in agriculture probably plays a role, as this sector is one of the major employers of women (Mehrotra and Parida 2017). As employment in agriculture declines, many women leave the labor force while others shift to the construction or manufacturing sectors, which do not offer conditions for stable long-term employment for women (Mehrotra and Parida 2017). Another factor is gender discrimination and social norms, which restrict women’s ability to enter or stay in the labor force (Desai and Jain 1994; Prillaman et al. 2017).

The role of marital status in determining women’s access to employment is an important way through which gender norms could impact female LFP. In rural areas, unmarried women are less likely than married women to participate in the labor market due to social restrictions and security concerns (Mehrotra and Parida 2017). Urban areas have the opposite phenomenon. Married urban women are less likely to participate in work outside the home relative to unmarried women due, perhaps in part, to normative views that married women should invest only in homemaking. Interestingly, the fall in employment rates has only occurred among married women in rural India (Afridi, Dinkelman, and Mahajan 2018). This may be happening because married women in rural areas have increased their investment in household work, mimicking the behavior of urban women as their household incomes have increased. While a working wife is undesirable to many men in India, the demand for an educated wife appears to be growing. Scholars have found a negative relationship between the expansion of education and female LFP (Chatterjee, Desai, and Vanneman 2018). Some have suggested that education is being used by young women and their families to improve their marriage prospects rather than as preparation for the workforce. Evidence of this is found in the fact that women with low and even medium levels of education are increasingly less likely to be matched with high-quality men on the marriage market (Klasen and Pieters 2015).

Across various settings in India, however, widowed women in working ages are observed to have one of the highest rates of employment relative to women of other marital statuses (Das 2015; Chen and Dreze 1992). In fact, a study of 134 countries found an association between a growing widowed population and rising female LFP due to the strong global pattern of high LFP rates for widows (Clark, York, and Anker 1999). India, which has the largest widowed population in the world, has not seen this rising female LFP primarily due to trends in falling LFP among the substantially larger population of married women.

The relationship between marriage and women’s engagement with the workforce has also been examined in a quasi-experimental setting. Jensen (2012) found that a program to increase the employment opportunities for young unmarried Indian women could lead to greater investment in their human capital and a delay of marriage and childbearing. Women who participated in the program expressed higher career aspirations than those who did not. These findings show that gender norms associated with marriage play an important role in limiting Indian women’s work participation.

### 2.2 Widowhood in India

In the Indian context, widows face unique vulnerabilities that may vary by caste, age, social context, and household composition. Historical evidence suggests that widows have often been the victims of social ostracism, enforced dress and behavior codes, physical and sexual violence, and accusations of witchcraft (Chen 2000). Indian feminist scholars have described widowhood cultural practices as a form of “social death” imposed to control a woman’s sexuality and “deny her personhood” since her identity independent of a man is considered a threat to the patriarchal system (Chakravarti 1998: 64).

There are reasons to believe that the status of widows may vary by caste. This is because of the complex ways in which caste and gender are intertwined in Indian society. Within the Hindu caste system, the social regulation of the behavior of women, including widows, serves as an “index for ascribing higher rank within the caste system” (Chakravarti 1998: 1). The strictest social regulation of widows is said to be among the highest caste, particularly the priestly communities of Brahmins. Brahmin and other upper caste widows were traditionally not permitted to eat non-vegetarian food, to eat from the same hearth as other family members, to wear jewelry, or to wear colorful clothing (Lamb 2000; Chen 2000). Widows are expected to live the remainder of their lives in austere remembrance of their husbands. This includes an expectation to remain outside of the labor force. Any deviations from this behavior may invite accusations of immorality or even accusations that the woman played a role in her husband’s death (Lamb 2000). Recent evidence has suggested that these diet restrictions and seclusion practices can have real implications for the physical health of Brahmin widows who were found to have BMIs comparable to women from the most marginalized caste groups, despite significantly higher household overall consumption (Jensen 2005).

For lower caste (Dalit) women, the death of their husband is likely to lead to less new social restrictions on their behavior than those placed on Brahmin women (Chen 2000). Dalit communities, where women are often wage earners, usually do not have the luxury of wasting the productive capacity of the women in their household through seclusion and withdrawal from the labor market. However, no study to date has systematically examined how the experience of widowhood varies by caste in India using panel data. Studies in the United States have shown that race and ethnicity moderate widowhood effects, suggesting that widowhood may be experienced differently by members of different social groups (Elwert and Christakis 2006; Angel, Jiménez, and Angel 2007).

There are also important age and life-course dimensions of widowhood. As mentioned earlier, a significant number of young and middle-aged Indian women are widowed. Historical evidence shows that the impact of widowhood is stronger when it occurs at an early life stage (Vignes 2017). The high mortality risks associated with young widowhood in some historical contexts may be mediated by household-level factors such as the number of young children (Alter, Dribe, and Van Poppel 2007). Young widowhood may place women in an especially precarious position in India compared to other social contexts. Whereas “women who are widowed after menopause and other widows once they pass menopause are often able to command significant respect, deference, and authority,” a young widow may be viewed as dangerous for the honor of the household (Chen 2000: 186).

Social context also plays a role in how widowhood will impact women. Women may experience widowhood differently depending upon which region of India they live in and whether they live in a village or a city. Researchers have long recognized that cultural norms in the southern states of India are quite different from those in the North, and these differences have implications for the status of women (Dyson and Moore 1983; Rahman and Rao 2004; Malhotra, Vanneman, and Kishor 1995). Marriages in South India are much more likely to be consanguineous, with women often marrying a cousin or uncle (IIPS and ICF 2017; Karve 1968). In such a context, widowed women in the South may have more social protections because of the proximity of their natal kin. In fact, it has been found that widows in southern states are more likely to inherit land from their husband than in many North Indian states (Agarwal 1998). Furthermore, research has suggested that the gap between the wellbeing of widows and non-widows was smaller in regions with a female labor-intensive crop (Jensen 2005). This could explain why widows in North India, where the main crop is wheat which utilizes more male labor, may have lower relative social position. Research on the marginalization of Indian widows primarily focuses on rural areas, where norms on widow seclusion and the stigma associated with widowhood are expected to be greater than in urban areas (Chen 2000; Chen and Dreze 1992; Drèze and Srinivasan 1997; Rahman, Foster, and Menken 1992). Less is known about the status of widows in urban areas.

Familial support and household composition are central predictors of the physical and mental health status of widows in India as in other settings. There are reasons to believe that these factors may also play a role in how widowhood impacts the LFP status of widows. The patrilocal and patrilineal marriage system in much of India often results in widows being cut off from their natal place. When a daughter leaves her father’s home after marriage, she may be viewed as having lost her rights to inheritance, as she is seen as a member of her husband’s family rather than her father’s (Agarwal 1998; Das Gupta 1995). Therefore, it is less common to find widows receiving significant support from their own kin. However, after the death of her husband, she may also be ostracized and seen as an outsider in her husband’s village (Chen and Dreze 1992). In either place, she may be expected to ‘earn her keep’ by contributing to the household financially when she may not have worked for wages before widowhood (Chen 2000). Previous research has highlighted that widows rarely receive financial assistance from their in-laws and often face harassment from them (ibid). Despite this mistreatment, survey work in the early 1990s found that the majority of rural North Indian widows continue to reside in their husband’s village after his death rather than returning to their village of origin (Chen and Drèze 1992).

Intra-household inequalities are an important cause of the higher mortality rates among widows in north India, which Chen and Drèze (1992) estimated to be three times that of married women. The highest mortality risk is associated with widows who live alone or in households headed by ‘others,’ anyone other than themselves or their son (Chen and Drèze 1992; Rahman, Foster, and Menken 1992). There is a widespread cultural expectation that adult sons should provide for the financial needs of their widowed mothers (Golandaj, Goli, and Das 2013). The power that a widow exercises in the household depends greatly on the support and respect she receives from her son, who often sets the tone that others in the household follow (Agarwal 1998). Coresidence and economic dependence on adult children, beyond being a social ideal, is likely also an economic necessity for elderly Indian widows. Globally, patterns of widow coresidence with their adult children tend to be higher in social contexts like India, which lack large state-funded social welfare and insurance programs for the elderly. Historical data from the United States shows that the establishment of Social Security is responsible for the majority of the massive trend in the expansion of elderly widows living alone between 1940 and 1990 (McGarry and Schoeni 2000).

While a large portion Indian widows are economically dependent on their adult children, many widows are financially independent, especially those who become widows earlier in life. On average, widowhood is generally associated with decreased employment for men and women, a trend that is shown to mediate the negative relationship between widowhood and health (Pandey and Kumar 2012). The economic shock following the loss of a breadwinner, however, may push some female widows into the labor force. Other widows may make a choice to enter the labor force after their husband’s death because they no longer face restrictions on their ability to work.

Entering the labor force or increasing one’s engagement in the labor force is not an easy transition for most widows. They may face social stigma or become the victim of malicious gossip for working outside of the home or not adhering to expectations of ritual mourning (Chen 2000). Some women face a bitter struggle to obtain the right to own their husband’s land (Agarwal 1998). Even if they keep the land, they may face difficulty in maintaining the farm due to a lack of social capital and business skills. Social taboos on women using the plow also affect widows who would like to manage the family farm after their husband’s death. Women who inherit a family business may face similar challenges in addition to experiencing discrimination from customers, suppliers, and employees. Obtaining a loan without a man to provide collateral on their behalf is another struggle for widows and is one of the reasons why widows have often benefitted the most from microfinance schemes (Cheston and Kahn 2002). Perhaps due to the challenges widows face as earners, widow-headed households are the poorest in India, with single widow households not far behind them (Drèze and Srinivasan 1997).

## 3. Research questions

This study will be the first, to the author’s knowledge, to examine how widowhood impacts Indian women’s labor force participation utilizing a panel dataset that tracks widows before and after the death of their husband. The analysis documents how the widowhood effects on work participation vary by age, caste/religion, relationship to household head, residence type (rural or urban), and region of India (North or South). In addition, the analysis will examine what types of occupations widows take on when they enter the labor force to uncover whether widows are employed in the same industries and jobs as married women.

The previous literature would suggest that widowhood effects on LFP will be highly stratified. Younger widows may be the most likely to see a positive widowhood effect on their work participation because they may not yet have adult sons on which they may rely financially. Their entry into the labor force may therefore be a necessity to support their family (Drèze and Srinivasan 1997; Chen 2000). Women who become widows in the oldest ages are not expected to see a positive widowhood effect on their work participation because they may be sheltered from the economic shock or unable to work due to their advanced age.

Widowhood effects may also be stratified by caste. A large literature in anthropology and related disciplines has documented the weak position of upper caste, particularly Brahmin, widows. According to this literature, strong norms of ritual mourning and seclusion keep many upper caste widows out of the labor force after the death of their husband (Lamb 2000; Jensen 2005).

Previous studies have suggested that living under the care of one’s adult children can have protective effects for Indian widows (Rahman, Foster, and Menken 1992; Golandaj, Goli, and Das 2013; Samanta, Chen, and Vanneman 2015). It is likely that widows who reside with their in-laws after the death of their spouse will have a positive widowhood effect on their work participation. Those who live with their adult children are not expected to observe any widowhood effects. Further, positive widowhood effects may be larger in North India and in rural areas where widows may be in a more vulnerable position relative to non-widows.

## 4. Empirical strategy

### 4.1 Data

Data from the India Human Development Survey (IHDS) was used in the analysis. The IHDS is one of the first large-scale nationally representative panel studies in India (Desai, Vanneman, and NCAER 2012). Using stratified random sampling, the survey covered 1,503 villages and 971 urban blocks located in 276 towns and cities (Desai, et al. 2010). Wave 1 was completed in 2004–2005 and Wave 2 was completed in 2011–2012. With a re-contact rate of 83%, the survey allows tracking of households as well as ‘split households’ within the same area. The individual data file used in the analysis contains information on all individuals whose information was collected in the household roster. The analysis is restricted to women present in both waves of the survey. Women who were unmarried, separated/divorced, or married but had not moved in with their husband yet (a practice known as *guana)* in either survey wave were dropped from the sample. As a result, the sample only includes those women whose marital status is either widowed or married in both survey waves so that the analysis can focus on the transitions between these marital statuses. Divorce and separation, the other means through which women could leave their marriage, are very uncommon in India. Dropping divorced or separated women reduced the sample by less than 1%. With no further restrictions, this leaves a final sample of 44,945 women used in the analysis. Because the household roster individual data is used, it is possible for more than one woman to be included in the sample per household.

### 4.2 Measures

This paper will focus on the process of widowhood, which is defined as changing one’s marital status from married to widowed. A time-variant dichotomous variable captures whether the respondent is widowed or married in IHDS I and II. This is the main independent variable. Of the 44,945 women in the sample, 3,217 women became widowed between the survey waves. There are 5,098 women who are listed as widowed in both waves of the survey and 36,558 whose status as married did not change. A small number of widows (72 women) in IHDS I remarried by IHDS II, and these women are included in the sample for regression analysis. The number of remarried widows is small perhaps because of taboos on widow remarriage. It is also possible that some women who remarried were lost to follow-up between the survey waves if they moved into a new household after remarriage.

The dependent variables in the analysis are labor force participation and the number of days worked in the past year. Labor force participation is a binary variable defined as working at least part-time over the past year, by completing a minimum of 240 hours of work in the year. The work data for the IHDS is collected from the income section of the household questionnaire, where detailed information was gathered on each household member’s participation in income-generating activities and their level of participation. The outcome variables capture various types of work, including (1) family farm work, (2) salaried work, (3) agricultural wage work, and (4) non-agricultural wage work. In IHDS II, there is a separate category of non-agricultural wage work documenting participation in the Mahatma Gandhi National Rural Employment Guarantee Scheme (NREGA), a government social security scheme that guarantees 100 days of work to every rural person wishing to participate. This program started nationally in 2008 between IHDS I and IHDS II. The high rates of participation in this scheme by women and the positive effect this has had on their status in the household has been documented by other studies (Desai, Vashishtha, and Joshi 2015). The independent variable used in the regression analysis is a continuous measure of the number of days worked in the past year. This variable has possible values ranging from 0 to 365.

A set of time-invariant moderation variables are included in the fixed effects regression analysis to show how the widowhood effects vary by different groups. The moderation variables include age in IHDS I, caste, relationship to the head of household in IHDS II, residence type, and region. Age in IHDS I ranges from 7 to over 100 in the sample. In the regression models, age in IHDS I is divided into ten age groups. The caste/religion variable combines information about the individual’s caste and religion using what was reported during IHDS I. The categories include the marginalized communities of Dalits or Scheduled Castes (formerly known as ‘Untouchables’), Adivasis or Scheduled Tribes, and Hindu Other Backward Classes (OBCs), a heterogenous group of non-Dalit castes recognized by the Indian government as socially and educationally disadvantaged. Brahmins are separated from other upper caste Hindus in order to test whether Brahmin women experience widowhood differently as the literature would suggest. The final categories in the caste/religion variable identifies Muslims and members of other minority religious communities (Christian, Sikh, Jain, etc.), which may have different widowhood norms than the Hindu caste groups. A categorical variable captures the relationship to the head of household listed in the survey roster in IHDS II after the woman would have already become widowed. The categories of relation to household head include (1) mother, (2) daughter-in-law, (3) other relation, and (4) self. In the last category, the widow is herself listed as the household head. A dichotomous variable indicates whether the respondent lives in an urban area as defined by the 2001 Indian Census. Another dichotomous variable divides the sample into North and South India. South India includes the states of Tamil Nadu, Pondicherry, Andhra Pradesh/Telangana, Karnataka, and Kerala. The remaining states are categorized as North.

The fixed effects methodology used in this analysis only examines variation within individuals, so fixed individual and household characteristics that do not change over time are controlled for in the analysis. Controls were added only for other time-variant characteristics that could also have an association with work participation. Controls are added for the number of days that the respondent was ill in the past year and the number of children in the household, both of which could impact her work participation. The regression models also include dummy variables indicating that the woman receives a widow pension or an old age pension. These two social security schemes could help mitigate the economic shock associated with widowhood and are expected to have a negative relationship with days worked in the past year. According to the IHDS II, 22% of widows are receiving a widow pension while 19% of elderly women receive an old age pension (Dreze and Khera 2017).

### 4.3 Methodology

The first set of results consist of descriptive statistics from the data on widowhood and employment from the IHDS I and II. This will be followed by results from fixed effects regressions that capture the widowhood effect on work participation measured by days worked in the past year. The IHDS provides the first opportunity to systematically examine the shock of widowhood and its implications for work participation by tracking women from before to after the death of their spouse. The advantage of the fixed effects approach is that each individual woman is treated as her own control. In the analysis that follows, the widowhood effect is the effect of a change in widowhood status on subsequent changes in women’s labor force participation. The fixed effects approach focuses only on within-individual variation, and therefore the widow coefficients presented in the regression tables are based on the women who experienced the transition into or out of widowhood between IHDS I and IHDS II. Fixed effects models do not include between-individual variation. Between-individual variation, such as comparing women of different marital statuses, may be confounded with unobserved characteristics of the respondents, thus introducing bias into the measures (Allison 2009). Fixed effects regressions are considered more robust because they eliminate this form of bias. This method is especially attractive for examining changes from a life-course perspective because it focuses on the changes related to events within an individual’s life rather than comparing across individuals in a cross-sectional analysis, as has been done in previous studies on widowhood.

The fixed effects regressions used in the analysis measure the number of days worked in the past year, *Y*_*it*_, through the model

$$Y_{it}=\alpha_{i}+{\text{Caste}}_{i}\theta +\beta_{1}{\text{Widow}}_{it}+\beta_{2}\left({\text{Caste}}_{i}*{\text{Widow}}_{it}\right)+\sum_{k}\beta_{3k}{\text{Controls}}_{itk}+t\;\beta_{0}+u_{it},$$

where the parameter *α*_*i*_ is an individual-specific constant that captures time-invariant characteristics of individuals and *tβ*_0_ is a time trend. The fixed effect model computes the difference ΔY_it_ = *Y*_*i*2_ − *Y*_*i*1_ between the two time periods and runs the regression

$$\Delta Y_{it}=\beta_{0}+\beta_{1}\Delta {\text{Widow}}_{it}+\beta_{2}\left({\text{Caste}}_{i}*\Delta {\text{Widow}}_{it}\right)+\sum_{k}\beta_{3k}\;\Delta {\text{Controls}}_{\text{itk}}+u_{it}.$$

The difference gets rid of the individual specific parameter *α*_*i*_ and the main effect of caste, the time-invariant moderator variable (Allison 2009). Therefore, the regression picks up the time-varying characteristics of widowhood, the controls, and the interaction between caste and the change in widowhood status. The caste interaction shows how the widowhood effects vary by different caste/religious groups in the sample. The same model is used with the other time-invariant moderating variables of age in IHDS I, relation to head of household in IHDS II, residence type, and region. Robust standard errors are used in the individual fixed effects analysis.

## 5. Results

### 5.1 Descriptive results

Table 1 provides some descriptive statistics comparing women who remained married in both survey waves, women who became widowed between IHDS I and II, and women listed as widowed in both waves. Recently widowed women are significantly older than the women who remained married. Their average age in IHDS II was 58 compared to 43 for the women who remained married. The women who are widowed in both waves are even older, with an average age of 65 in IHDS II. Belonging to an older age cohort, it is not surprising to find that the recently widowed women are less likely to be literate than the women who remained married (35.4% literate compared to 50.3%). Households with a widow were no less likely to be poor, though they did have fewer workers and fewer children present in the household. Previous research has also shown that households in which widows lived but did not head were no poorer than other households (Drèze and Srinivasan 1997).

After the death of their spouse, 9.1% of recently widowed women were living alone. Though efforts were made by the IHDS team to follow ‘split’ households, it is possible that some recently widowed women who changed residence after the death of their husband may leave the sample leading to an over-representation of widows living alone. This could happen for widows who moved in with other family members outside of the sampling region after the death of their husband. However, the inclusion of women who were already widowed in IHDS I in the analysis allows for an examination of a greater array of post-widowhood living arrangements, including women who may have moved after widowhood. In IHDS I, 5.4% of the widowed women were living alone, but by IHDS II, 8.8% of them were living alone. The new widows have similar rates of living alone to those who were already widowed in Wave I, suggesting that the sample of new widows is probably not severely overrepresenting widows who remained in place after widowhood. The large rate of living alone for both widow groups is a cause of concern given the advanced age of this population. Studies suggest that there may be adverse health effects associated with living alone in old age (Golandaj, Goli, and Das 2013; Sudha et al. 2007; Chen and Dreze 1992; Samanta, Chen, and Vanneman 2015).

The deceased husbands of the new widows died, on average, at around age 61. They were less likely to be literate and more likely to be listed as the head of household than the husbands who did not pass away between the survey waves. These men had low labor force participation rates, which is not surprising given their age. Of the husbands who died between the panel waves, 30.5% were not in the labor force in IHDS I compared to only 8.2% of those husbands who did not perish. For the younger new widows (those who were 45 or younger in IHDS I), only 7.9% of their husbands were not in the labor force in IHDS I.

Labor force participation by marital status is displayed in the lower portion of Table 1. Newly widowed women experienced some large changes in their LFP between the survey waves. The largest effect seems to be in salary work, where new widows have a participation rate of 6.8% by IHDS II. By 2011–2012 the new widows look like the widows in 2004–2005, though these widows have decreased their participation in most categories by 2011–2012. This may be due to the advanced age of the already widowed women. For both waves, married woman had higher rates of participation in farming than women of the other marital statuses, an observation which matches with the findings of other scholars (Mehrotra and Parida 2017). Table 1 is limited in what it can tell us about the different rates of participation by marital status because it does not incorporate the significant age difference of the widowed and married populations.

### 5.2 Age-specific labor force participation rates

**Figure 1:**
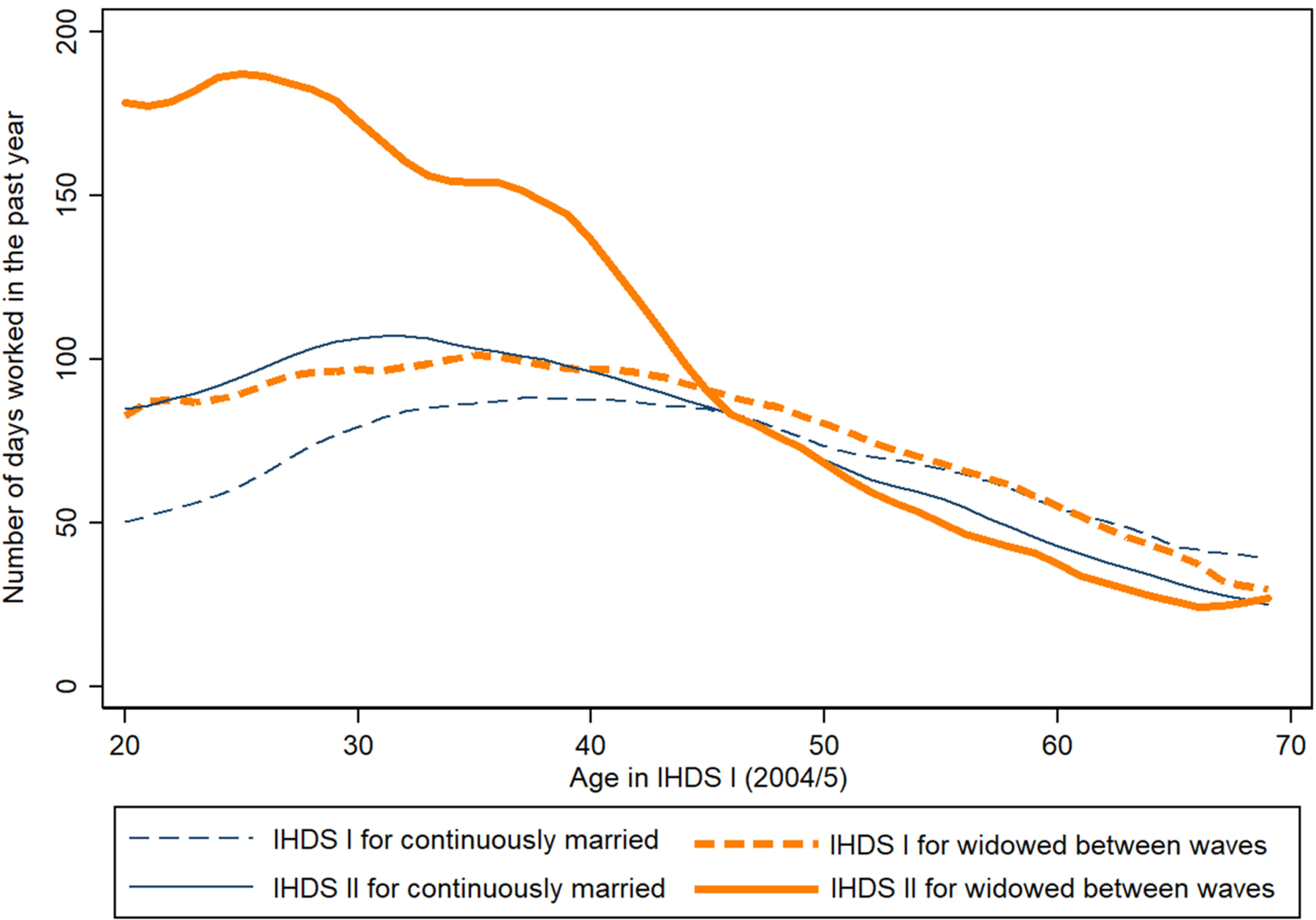
Average number of days worked in the past year by widowhood status, survey wave, and age *Note:* Lines plot the local polynomial regression line fitting mean days worked in the past year by age in IHDS I for women of different widowhood statuses. *Source:* India Human Development Survey I and II, 2004–2005 and 2011–2012.

Figure 1 shows the average number of days worked in the past year by age of respondent in IHDS I with a plotted local polynomial regression line. Plotted on the same graph are the women’s days worked in IHDS I (2004–2005) and IHDS II (2011–2012). The new widows (‘widowed between waves’) have low average number of days worked in the 2004–2005 survey wave when they were still married. Their participation mirrors that of the other married women, though those who become widowed at younger ages do work slightly more even before their husband’s death. We can interpret this gap between the young women who will and those who will not become widows as driven by selection. Younger women who eventually become widows (those younger than 45 in IHDS I) worked slightly more before widowhood than the average married woman in their age group. This gap narrows as the age of the woman increases. Women widowed before the age of 52 (which means they were aged 45 or younger in IHDS I, seven years earlier) may be selected from more disadvantaged households. Mortality rates for men are generally low in that age range (even if we assume that the husbands are older than their wives), so the death of these women’s husbands may have been unexpected. For men in this age group, it is more likely that they perished from preventable causes such as accidents/injury, infectious diseases, and self-harm than older men who tend to be taken by chronic and age-related illnesses. It should be noted that the number of women widowed in these earlier ages is not insignificant. Of the 3,217 women widowed between the survey waves, 30.1% (967 women) were aged 45 or below in IHDS I.

By IHDS-II the recently widowed women had increased their workforce participation by huge amounts for all women aged 45 and younger in IHDS I, as shown in Figure 1. We can interpret this large increase as the widowhood effect. The groups converge by around age 45 in IHDS I. This means that women appear to only be at risk for a large widowhood effect on their labor force participation if they became widowed before the age of 52 (the age that 45-year-old women would be by IHDS II). Both married and widowed women have low levels of participation in the labor force beginning in their mid-50s.

**Figure 2:**
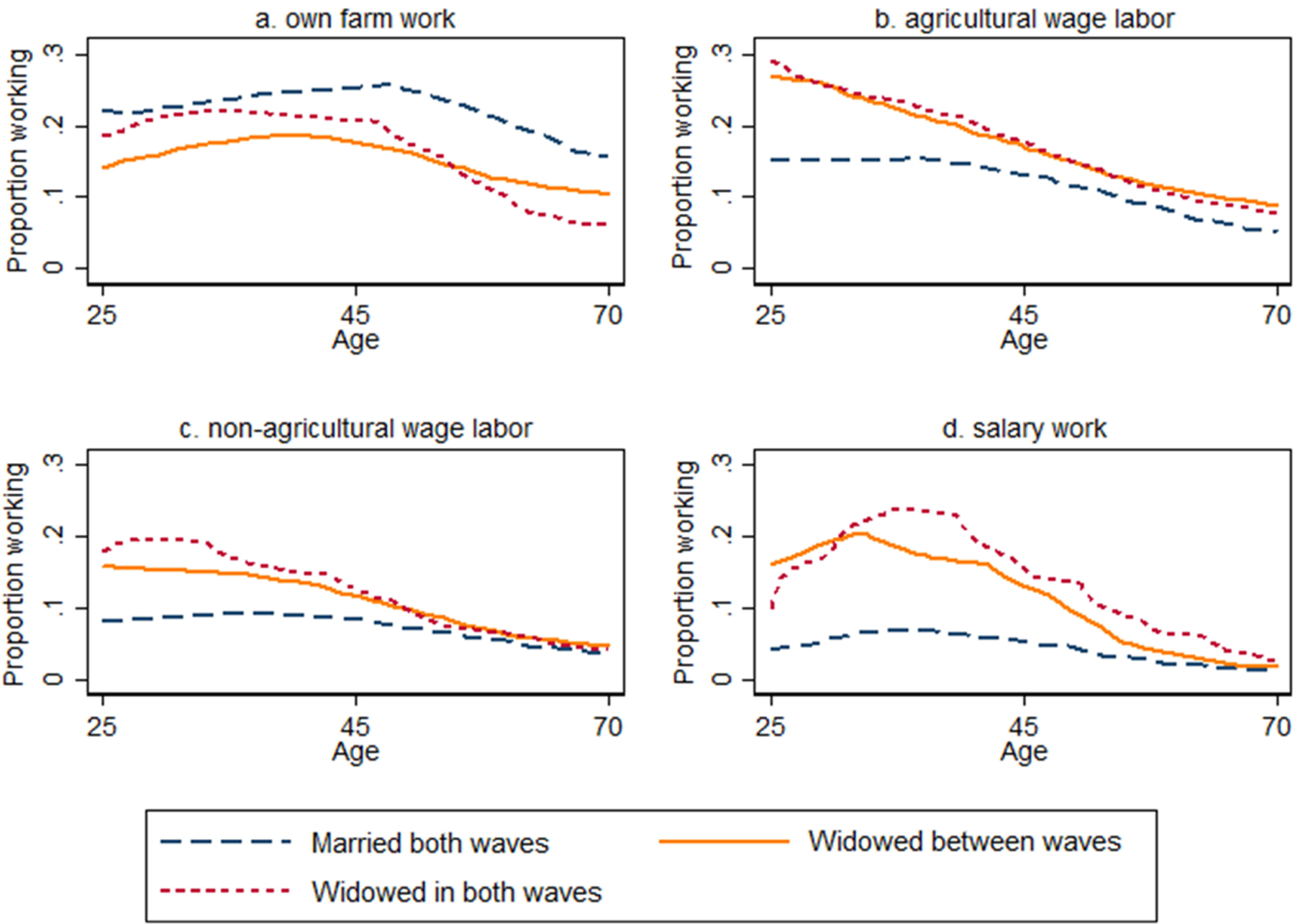
Labor force participation by type, age, and widowhood status (IHDS II) *Note:* Lines plot the local polynomial regression line fitting proportion in labor force by five-year age groups for women of different widowhood statuses. *Source:* India Human Development Survey I and II, 2004–2005 and 2011–2012.

Figure 2 depicts LFP rates in IHDS II by marital status and age broken into four broad categories of work. It confirms previous research showing that married women are more likely than widowed women to work on a family farm (Mehrotra and Parida 2017). This holds at every age. The largest gap between widowed women and women who remain married appears to be in salary work. Widows are more than twice as likely to be employed in a salaried job relative to married women at almost every age. Widowed women also have relatively higher participation rates in both agricultural and non-agricultural wage labor. Figure 2 also shows that the newly widowed sample has work participation behaviors in IHDS II, which are similar to those who were widowed in both waves of the survey.

### 5.3 Occupations of widows and married women

Details on occupations were collected by the IHDS and provide more insight into the work experience of new widows. The two most common industries of wage/salary employment for both married and widowed women are agriculture and construction. This accounts for 70% of all new jobs taken between the survey waves for married women and 59% of all new jobs for those women who were recently widowed. For the newly widowed women who joined the labor force, the next most common occupations after agriculture and construction labor are domestic service, food preparation, and clerical work. New entrants into the labor force who were married took similar jobs, though they had a higher rate of employment as teachers and a lower rate of employment as cooks, maids, or clerical workers. New widows who only recently joined the labor force in wage and salary work had average annual earnings of Rs. 25,259 compared to the married women who joined the labor force, who had earnings of Rs. 20,319. This may be due in part to the fact that widowed women who started paid work were less likely than married women to take positions that were casual or based on short contracts, suggesting a deeper and more secure connection to the labor force. Of all the newly widowed women who joined the paid labor force, 18.6% of them entered permanent positions, compared to 12.6% of the married women who entered the labor force. Married women were also more likely to report that they worked as a casual daily wage worker compared to widowed women.

Data on occupations shows that the government is a major employer of both married and widowed women. Many of the married women who joined the labor force between the two waves of the survey did so to participate in the Mahatma Gandhi National Rural Employment Guarantee Scheme (NREGA), a government social security scheme that guarantees 100 days of work to every rural person wishing to participate. Nearly a third of the married new labor force entrants did some work for NREGA in the past year compared to a quarter of newly widowed women who entered the labor force.

However, there was suggestive evidence that widows were employed by the government in more permanent ways and that they may be benefiting from preferential hiring for government jobs. Rather than receiving part-time causal employment through NREGA as many married women did, widows tended to work in more permanent government jobs, such as serving as a cook for the Mid-Day Meal scheme at a local public school, working as assistant or cleaner in a government office, or working as an *Anganwadi* (government crèche/health education center). Of all the newly widowed women who entered the workforce, around 13% got a regular government job whereas only around 8% of continuously married women entered these types of jobs. Many government bodies outline hiring guidelines that call for special preference to be given to widows seeking employment given their vulnerable social and economic position (*Times of India* 2019). One study found that one-fourth of all Mid-Day Meal cooks were widows in the state of Karnataka, at least partly due to preferential hiring for widows (Dreze and Goyal 2003). Furthermore, about one-third of newly widowed women who entered the salaried workforce had husbands who had worked in salaried jobs in IHDS I. These women may have benefitted from preferential hiring programs for the widows of previous employees or widows more generally. The findings from the IHDS suggests that many widowed women who enter the labor force find a permanent income source, often through government employment.

### 5.4 Fixed effect regression results

**Figure 3:**
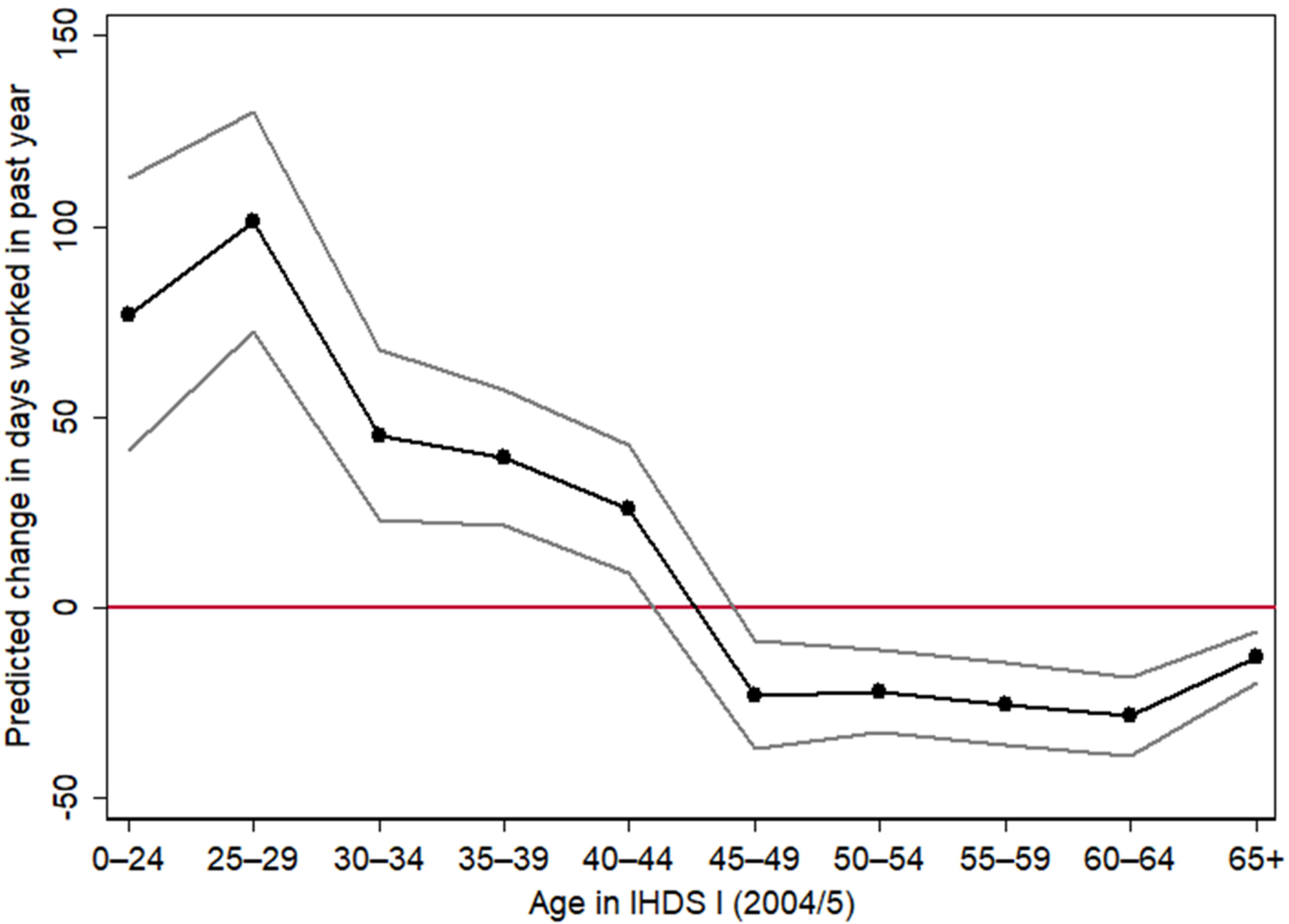
Predicted widowhood effects on days worked in the past year by age *Note*: Connected black dots plot the predicted widowhood effects from the individual fixed effects regression presented in Model 3 of Table 2. The gray lines show the 95% confidence interval. *Source*: India Human Development Survey I and II, 2004–2005 and 2011–2012.

Regression analysis provides a clearer picture of the LFP changes associated with widowhood. Results from individual fixed effects linear regressions can be found in Figure 3 and Tables 2, 3, and 4. The dichotomous ‘widow’ variable reflects the effect of a change from married to widowed between the survey waves. In Model 1 of Table 2 (the empty model), the coefficient for widowhood is negative though not statistically significant. This means that, without accounting for age of the woman, the shock of widowhood is not associated with any change in the number of days worked in the past year. The Wave II variable is significant, indicating that, on average, there was an increase in the number of days worked for the sample between the two waves of the survey, controlling for changes in widowhood status. The subsequent models in Table 2 reveal that the null result in Model 1 has occurred because it does not incorporate the important age dimension of widowhood effects.

Interacting the widowhood effect with the age group of the woman in IHDS I (a time-invariant variable) permits an exploration of how widowhood effects vary by the age at widowhood in Models 2 and 3. The reference age group in the regression is the oldest age bracket of women, aged 65+ in IHDS I. The age variable does not vary within each individual, therefore no main effect can be calculated for age. There is a large and statistically significant positive widowhood effect for women in the younger age cohorts. A positive widowhood effect means that the shock of widowhood is associated with an increase in the number of days that a woman reported that she worked in the past year. Starting in the 45–49 age group, widowhood had the opposite effect on days worked. Widowhood is associated with statistically significant decreases in days worked for all women who became widowed after reaching the age cohort of 45–49 in IHDS I. These results change little in Model 3 when the controls are added to the models.

Figure 3 plots the predicted widowhood effects by age calculated from Model 3 of Table 2. Like the descriptive results in Figure 1, there is a clear age pattern with a crossover in the 40s. For example, a woman aged 25 in 2004–2005 who was widowed by 2011–2012 would be expected to increase her days worked by 97 days per year (interaction + the main effect) including the controls for other changes (Model 3). This is a huge effect given that the mean number of annual days worked for a woman aged 25–29 in IHDS I was only 69 days. This means that a newly widowed woman in that age group works 141% as much as she did seven years previously, before the death of her husband. As shown in Figure 3, women in this age group face the largest widowhood effect. If instead she was aged 45 in 2004–2005 and became widowed by 2011–2012 when she was 52, then Model 3 predicts that widowhood would decrease her days worked in the past year by 23 days (interaction + main effect). For women who were 45 or older in IHDS I, widowhood is associated with a statistically significant decrease in the number of days worked in the past year and this effect is similar in magnitude for all older women, as illustrated in Figure 3. Widowhood is associated with both decreases and increases in work participation. The direction of the effect depends on the age at which a woman becomes widowed.

The coefficients for age interactions change little when controls are added in Model 3 of Table 2. The coefficient for ‘days sick’ shows that for a one-day increase in the number of days that a respondent was suffering a major illness in the past year, the number of days she worked is expected to decrease by only .037. The coefficients for the widows’ pension and old age pension dummy variables indicate that a household obtaining these pensions between the waves of the survey was associated with a decrease in days worked in the past year. These significant coefficients suggest that these government schemes may suppress work participation by providing economic protection for widowed women. When the number of children aged 0–14 in the house increases, there is a corresponding decrease in days worked for the women in the sample. Both the days sick and the children variables have effects that are relatively small in magnitude.

The next set of regressions, in Table 3, examine how widowhood effects on days worked vary by caste and the widow’s relationship to the head of household. Since the widowhood effects were shown to be different for women widowed before age 52 in IHDS II (45 in IHDS I) relative to those who became widowed at a later age, the sample was split into a younger and older age group. In the first set of models, the aim was to test the hypothesis that upper caste, especially Brahmin women, are less likely to experience a positive widowhood effect on their work participation because of strict norms of widow seclusion and ritual mourning. The widow variable is interacted with the time-invariant variable, which indicates the caste or religious community of the woman. There is no main effect of caste/religion because the fixed effects regression cannot compute a coefficient for variables that do not change over time. Results presented in Models 1, 2, and 3 suggest that widowhood effects vary only slightly by caste or religious community.

Table 3 provides some suggested evidence against the idea that Brahmin and other upper caste women are more likely to withdraw from the labor force after widowhood. The coefficient for Brahmin women in Model 3 reveals that older Brahmin widows experience a marginally significant positive widowhood effect on the number of days worked in the past year. Brahmins are the only group that experience a positive widowhood effect on work participation in older ages that approaches statistical significance. This finding is contrary to the expectation, which posited that Brahmin women may be the most likely to face social pressure to perform ritual mourning and social seclusion after the death of their spouse, which would remove them from the labor force (Lamb 2000; Chen 2000; Jensen 2005). For the women aged 52 or younger (45 in IHDS I), there are no statistically significant differences in widowhood effects between Brahmins (the reference group) and women from other communities. Predicted widowhood effects calculated from Model 2 on the younger woman group reveals that other upper caste, OBC, and Dalit women all experience statistically significant positive widowhood effects. Overall, there is very little evidence that caste or religious identity is an important moderator of how widowhood impacts a woman’s labor force participation.

Models 4, 5, and 6 of Table 3 examine whether household composition moderates the relationship between widowhood and women’s employment. This is accomplished by interacting the widowhood variable with a time-invariant categorical variable indicating her relationship to the head of household in IHDS II. Based on previous research, women who were the mothers of the head of household were expected to have no or negative widowhood effects, and those who were daughters-in-law were expected to have a positive widowhood effect. The sample is again split into an older and younger sub-sample for Models 5 and 6 given the observed age pattern of widowhood effects.

Recently widowed women who live with their in-laws saw large and statistically significant positive widowhood effects on their work participation as shown in Models 4 and 5. Only a small number of women in the older age group are listed as living in a household headed by their in-laws, so it is not surprising that the coefficient does not reach significance in Model 6. As expected, living in a household headed by one’s children has a protective effect for widows, preventing widowhood from compelling women to enter the labor force. Being the mother of the head of household was associated with a negative and statistically significant widowhood effect for older widows. Few women in the younger age group were the mother of the head of household. Younger women were more likely to live with their in-laws or in households headed by themselves. These statuses were associated with statistically significant increases in the number of days worked as shown in predicted widowhood effects calculated from Model 5. The predicted widowhood effects calculated from Model 6 only reached statistical significance for mothers of the head of household and this relationship was negative. The negative widowhood effect in older ages is driven almost entirely by women who reside in a home headed by their adult children.

Table 4 shows how context moderates the way that widowhood will impact both older and younger women. Models 1, 2, and 3 of Table 4 depict the interaction of widowhood with region in individual fixed effects regression models. Model 2 reveals no statistically significant difference between North and South India in terms of how widowhood impacts younger widows. There were differences, however, for the older age group. In South India, widowhood was associated with a statistically significant decrease in the number of days worked in the past year for women aged 45 and older in IHDS I. For widows in North India, there was essentially no effect on days worked in the past year associated with widowhood for older women. This fits with previous studies that have highlighted the especially vulnerable position of widows in the north of India (Chen and Dreze 1992).

Models 4, 5, and 6 in Table 4 examine how widowhood is experienced differently in rural and urban areas. On average, rural women work more days than urban women in India. However, widowhood was associated with a larger increase in days worked for urban widows than rural widows as shown in Models 4 and 5. Urban women who become widowed before age 52 are expected to increase their annual days worked by 59 days (main effect + interaction), which is a 34% increase in the annual mean days worked. There was no statistically significant difference in the widowhood effects experienced by rural and urban women widowed at older ages. The results in Table 4 suggest that context plays a role in determining whether widowhood will increase a woman’s engagement with the labor force.

## 6. Discussion

This paper has been the first to examine the impact of widowhood on Indian women’s labor force participation using panel data that is able to track women before and after the shock of widowhood. Descriptive statistics and fixed effects regression results reveal a strong age pattern to the way that widowhood impacts a woman’s work participation. Women who were widowed before the age of 52 saw a large increase in the number of days they worked after the death of their husband. For women widowed after the age of 52, widowhood has a negative impact on the number of days they worked. Consideration of the life course gives some possible explanations for why the widowhood effect on women’s LFP has a crossover in her early 50s. After age 52, many women would have a son at a working age who may provide her financial support. Young widows are especially vulnerable to the shock of widowhood because their husband was more likely to be the breadwinner of the household than the husbands of older widows. They are also more likely to have young children who are still financially dependent. Therefore, losing a spouse is more likely to be accompanied by an economic shock for younger widows relative to those who lose their husbands in old age.

Data on the industries and occupations that women work in after widowhood reveals distinct trends. Compared to women who remain married, widowed women are less likely to work in the NREGA work guarantee program, on a family farm, or in casual jobs. It is possible that widowed women become alienated from family enterprises upon the death of their spouse, as has been suggested in previous literature (Chen 2000). Given that there may be a battle over inheritance of the family land or that they may struggle to maintain land that is transferred to them, it is not surprising to find that rates of family farm work are lower for widowed women. Instead, widowed women are more likely to be employed in non-agricultural wage labor and salaried work. They also have higher income than married women entering the labor force because they are more likely to be employed in a full-time permanent position. There is some evidence that widows may be benefitting from preferential hiring programs through the Indian government.

Results from individual fixed effects regressions presented in Tables 3 and 4 show how widowhood effects are experienced differently by women of different castes/religions, household living arrangements, and social context. The literature on widowhood in South Asia has often emphasized the relationship between caste and widowhood (Chen 2000; Lamb 2000). Increased seclusion and norms on ritual mourning are expected to be central parts of the widowhood experience within upper caste communities. In this study, however, Brahmin women were the only group to have a positive widowhood effect for women aged above 52. It is possible that, despite norms on ritual mourning and seclusion, Brahmin women are still forced to enter the labor force after widowhood because of economic necessity. An alternative possibility is that norms have changed in the Brahmin community since foundational work on widowhood was published in the 1990s and early 2000s. Perhaps norms on the necessity of widow seclusion have faded and Brahmin women now face fewer restrictions on their employment. Overall, however, caste or religion do not appear to be an especially important moderator of the widowhood effect. There were relatively similar trends across different social groups.

The experience of widowhood differs more dramatically by post-widowhood living arrangements. Older women who were the mother of the head-of-household in IHD II decreased their labor force participation after widowhood while younger women who were the daughter-in-law of the head of household increased their participation by a large amount. This data suggests that a widow’s economic dependency is often transferred from her husband to her son after her husband’s death. A widow’s in-laws may not be able or willing to provide for her financially. These findings build on a robust literature on the importance of household composition in determining women’s status in the household in South Asia (Debnath 2015).

Contextual factors also moderate the effect of widowhood on women’s work participation. It was in urban areas, where women’s work participation is lowest, where the widowhood shock had a larger impact on younger women’s engagement with the labor force. There was also further evidence of the especially vulnerable position of older widows in North India. It was only in South India that widowhood was associated with a decrease in the number of days worked for women widowed after the age of 52. Older South Indian women, many of whom are in consanguineous marriages, could benefit from greater economic protection after widowhood provided by their family and kin networks.

The individual fixed effects models employed in the analysis are advantageous because they remove much of the omitted variable bias that is present in regular regression modelling. However, as with all research, there are some limitations to the analysis. It is always possible that some key time-variant variable has been omitted from the model. This analysis is also constrained in its ability to uncover the mechanisms and causes of the associations documented. Entry into the labor force could signal economic distress after the loss of the household’s main breadwinner. At the same time, widowhood could also mark the relaxation of some control over her behavior, allowing her to finally pursue her desire to enter the labor market. Future research is needed to help parse out which mechanism best explains the trends observed in this paper and to uncover what employment means to these widows.

Widows have regrettably received limited attention in the literature on female labor force participation, despite having among the highest work participation of any marital status group. Around 7.4% of Indian women are widows and, unlike in other social contexts, they are not overwhelmingly found in the oldest age groups. In fact, a substantial portion of the working age female population are widows. For those women widowed during working ages, widowhood is associated with large increases in engagement with the labor force, some of which appears to be facilitated by government preferential hiring programs. This is an encouraging result, as it suggests that government interventions can be effective in expanding women’s access to stable permanent employment.

In addition, the findings of this paper give insights into Indian women’s relationship to the labor market by providing further evidence of the importance of marital status as a determinant of women’s work participation. Social norms about male breadwinning appear to play a large role in keeping married women out of the labor force. The absence of a husband or an adult son willing and able to financially support them pushes widows into employment. This appears to happen regardless of community norms on widow seclusion. Researchers should pay more attention to how gender norms associated with marriage suppress married women’s work participation.

## Figures and Tables

**Table 1:** Descriptive statistics on married and widowed women in IHDS I and II survey waves

		Married in both waves	Widowed between waves	Widowed in both waves
Literate (%)		50.3	35.4	25.9
Urban (%)		28.8	30.4	31.3
Reside in southern state^a^ (%)		21.3	26.4	29.6
Age	2004/5	36	51	58
	2011/12	43	58	65
Poor^b^ (%)	2004/5	22.3	22.8	22.7
	2011/12	17.5	17.6	18.8
Number of workers in household	2004/5	2.3	2.2	1.9
	2011/12	2.1	1.7	1.8
Number of children aged 0–14 in household	2004/5	2.1	1.6	1.6
	2011/12	1.6	1.3	1.2
Living alone (%)	2004/5	0.0	0.2	5.4
	2011/12	0.1	9.1	8.8
Living in two-person household (%)	2004/5	3.7	8.4	8.1
	2011/12	7.4	10.9	9.1
*Relation to head of household (%)*				
Wife	2004/5	69.9	80.8	-
	2011/12	80.3	-	-
Daughter-in-law	2004/5	24.0	4.3	2.6
	2011/12	12.6	2.9	1.1
Mother	2004/5	1.1	8.2	46.4
	2011/12	1.8	29.8	47.0
Self	2004/5	1.4	4.5	42.0
	2011/12	3.3	62.9	42.1
*Percent in labor force by type*				
Family farm	2004/5	23.4	18.7	12.1
	2011/12	22.8	12.6	8.3
Agricultural wage labor	2004/5	13.2	13.2	11.2
	2011/12	13.0	11.9	8.5
Non-agricultural wage labor	2004/5	3.8	3.8	4.8
Non-NREGA	2011/12	3.8	4.7	3.1
NREGA^c^	2011/12	4.2	3.2	2.4
Salary	2004/5	3.1	3.7	6.6
	2011/12	4.8	6.8	5.7
Family business	2004/5	2.9	3.3	3.1
	2011/12	4.3	3.3	2.8
Number of women		36,558	3,217	5,098

a Southern states include Andhra Pradesh/Telangana, Karnataka, Kerala, Tamil Nadu, and Pondicherry;

b Poverty defined by 2005–2011 Tendulkar cutoff;

c NREGA refers to the Mahatma Gandhi National Rural Employment Guarantee Scheme, a rural employment guarantee program run by the Indian government.

*Note:* This analysis only includes women who were present in both panel waves of the Indian Human Development Survey (IHDS), 2004–2005 and 2011–2012.

**Table 2:** Coefficients from individual fixed effects regressions on days worked in the past year by widowhood status and age

	Model 1	Model 2	Model 3
Widow	−3.766 (2.357)	−15.739** (3.313)	−13.147** (3.336)
Age in IHDS I^a^		-	-
*Age interactions with widow (reference: aged 65+ in IHDS I)*			
Widow × aged 0–24		89.986** (18.556)	89.781** (18.550)
Widow × aged 25–29		111.727** (15.368)	110.442** (15.456)
Widow × aged 30–34		60.449** (11.873)	58.298** (11.897)
Widow × aged 35–39		53.844** (9.610)	52.435** (9.628)
Widow × aged 40–44		39.999** (9.094)	39.161** (9.135)
Widow × aged 45–49		−9.680 (7.821)	−9.945 (7.831)
Widow × aged 50–54		−9.842 (6.378)	−8.954 (6.362)
Widow × aged 55–59		−13.336* (6.363)	−12.261+ (6.393)
Widow × aged 60–64		−16.356** (6.184)	−15.454* (6.195)
Days sick (past year)			−0.037** (0.011)
Gets widows pension			−8.023* (3.175)
Gets old age pension			−19.541** (2.769)
Number of 0–14 children in house			−1.125** (0.316)
Wave II	12.771** (0.602)	12.808** (0.601)	13.137** (0.625)
Constant	72.592** (0.403)	73.707** (0.367)	76.100** (0.722)
Number of women	44,945	44,945	44,945

a Main effect of age in IHDS I is absorbed by the individual fixed effect and cannot be separately identified because there is no variation over time.

*Notes:* Robust standard errors in parentheses;

** p < 0.01,

* p < 0.05,

+ p < 0.1

*Source:* Data from the India Human Development Survey (IHDS) I and II, 2004–2005 and 2011–2012.

**Table 3:** Coefficients from individual fixed effects regressions on days worked in the past year by widowhood status, caste/religion, and relationship to head of household

	Model 1	Model 2	Model 3	Model 4	Model 5	Model 6
	Full sample	<45 in IHDS I	45+ in IHDS I	Full sample	<45 in IHDS I	45+ in IHDS I
Widow	8.392 (8.763)	47.495 (28.920)	13.473+ (7.597)	64.584** (15.695)	53.262** (16.252)	74.676 (67.200)
Caste/religion^a^	–	–	–			
*Caste × widow interaction (reference group: Brahmins)*						
Other upper^b^ × widow	−8.359 (10.267)	−14.591 (31.763)	−11.507 (9.209)			
OBC^c^ × widow	−15.822+ (9.587)	−16.647 (30.146)	−24.718** (8.537)			
Dalit × widow	−2.335 (10.150)	5.073 (30.750)	−19.097* (9.116)			
Adivasi × widow	−14.287 (12.127)	−25.157 (31.974)	−27.933* (12.572)			
Muslim × widow	−11.691 (10.915)	−20.912 (32.779)	−14.334 (9.944)			
Other^d^ × widow	−6.164 (14.830)	−48.656 (39.128)	4.735 (15.167)			
Relation to household head in IHDS II^a^				-	-	-
*Relation to household head in IHDS II× widow (reference group: daughter-in-law)*						
Self × widow				−55.396** (15.989)	−16.242 (17.208)	−71.161 (67.298)
Mother × widow				−95.159** (15.991)	−56.539* (23.134)	−90.974 (67.275)
Other relationship × widow				−63.842** (26.881)	−30.450 (67.761)	−76.034 (67.753)
Controls^e^	X	X	X	X	X	X
Wave II	13.049** (0.625)	22.884** (0.775)	−8.059** (1.024)	13.053** (0.625)	22.880** (0.774)	−.997** (1.025)
Constant	75.363** (0.737)	76.403** (0.973)	67.295** (1.133)	76.226** (0.731)	76.387** (0.974)	68.206** (1.112)
Women	44,945	29,262	15,683	44,945	29,262	15,683

a Main effect cannot be separately identified because there is no variation over time.;

b Non-Brahmin upper caste Hindus;

c Hindus from Other Backward Classes;

d Jains, Sikhs, and Christians;

e Controls for days sick, receipt of widow or old-age pension, and number of children aged 0–14 in household.

*Notes:* Robust standard errors in parentheses;

** p < 0.01,

* p < 0.05,

+ p < 0.1.

*Source:* Data from the India Human Development Survey (IHDS) I and II, 2004–2005 and 2011–2012.

**Table 4:** Coefficients from individual fixed effects regressions on days worked in the past year by widowhood status, region, and type of residence

	Model 1	Model 2	Model 3	Model 4	Model 5	Model 6
	Full sample	<45 in IHDS I	45+ in IHDS I	Full sample	<45 in IHDS I	45+ in IHDS I
Widow	−8.543+ (4.518)	28.337** (9.703)	−13.372** (4.750)	−7.450** (2.752)	21.623** (6.126)	−6.574* (2.932)
Region^a^	–	–	–			
*Region × widow interaction (reference group: South)*						
North × widow	9.456+ (5.174)	8.066 (11.096)	12.252* (5.361)			
Residence type^b^				–	–	–
*Residence type × widow interaction (reference group: rural)*						
Urban × widow				19.114** (5.141)	37.502** (10.996)	7.599 (5.085)
Controls^c^	X	X	X	X	X	X
Wave II	13.043** (0.625)	22.888** (0.775)	−8.051** (1.024)	13.042** (0.625)	22.900** (0.775)	−8.024** (1.024)
Constant	75.389** (0.738)	76.411** (0.973)	67.376** (1.137)	75.356** (0.737)	76.407** (0.972)	67.254** (1.135)
Women	44,945	29,262	15,683	44,945	29,262	15,683

a Main effect of region cannot be separately identified because there is no variation over time. South included the states of Andhra Pradesh/Telangana, Karnataka, Kerala, Tamil Nadu, and Pondicherry. The rest of India was classified as North;

b Main effect of residence type cannot be separately identified because there is no variation over time;

c Controls for the number of days sick in the past year, receipt of widow or old-age pension, and number of children aged 0–14 in the household.

*Notes:* Robust standard errors in parentheses;

** p < 0.01,

* p < 0.05,

+ p < 0.1

*Source:* Data from the India Human Development Survey (IHDS) I and II, 2004–2005 and 2011–2012.
